# The Transfer of In-Game Behaviors and Emotions to Real-World Experiences in Game World

**DOI:** 10.3390/bs15091203

**Published:** 2025-09-04

**Authors:** Zhuoyue Diao, Pu Meng, Xin Meng, Liqun Zhang

**Affiliations:** School of Design, Shanghai Jiao Tong University, Shanghai 200240, China; diaozhuoyue@sjtu.edu.cn (Z.D.); mengpu0312@sjtu.edu.cn (P.M.); mx714540581@sjtu.edu.cn (X.M.)

**Keywords:** Game Transfer Phenomena (GTP), in-game behavior, emotional expression, virtual-to-real experience

## Abstract

This study investigates the complex interaction between in-game behaviors, post-game emotional expressions, and Game Transfer Phenomena (GTP) among MOBA players. A multidimensional framework is adopted to examine how gaming experiences shape real-world cognition, perception, and behavior through the integration of objective behavioral metrics and affective computing-based emotion recognition. The results indicate that in-game Deaths are negatively associated with altered sensory perceptions—specifically Altered Visual and Auditory Perceptions (AVP and AAP)—suggesting that performance failures may disrupt immersive engagement. In contrast, Assists, as indicators of team-based collaboration, are positively associated with Automatic Mental Processes (AMP), highlighting the cognitive impact of cooperative gameplay. Although no significant mediating effects were observed, emotional dimensions, such as Social Discomfort and Cognitive Focus, offered additional insights into the transfer between in-game and post-game experiences. These findings bridge the gap between virtual and real-world experiences, offering theoretical advancements in GTP research and practical implications for game design, emotional regulation, and psychological interventions.

## 1. Introduction

In recent years, the development of virtual worlds has been accelerated by substantial corporate investments in online and digital technologies, enhancing engagement among users and virtual ecosystems. The profound influence of the technological revolution on humanity has made it increasingly challenging to fully comprehend the extent of technology’s impact on society and individual lives (Volti & Croissant, 2024). Within this digital landscape, video games have emerged as a dominant form of virtual interaction, offering immersive multimedia experiences that deeply engage players (Guynup, 2012). Among these, Multiplayer Online Battle Arena (MOBA) games have become one of the most widely played genres. According to the 2024 China Gaming Marketing Trend Report (Fastdata, 2024), the number of gamers in China exceeded 700 million in 2023, with MOBA games dominating the mobile gaming sector. However, the deep engagement that video games foster has sparked interest in how such experiences might extend beyond the virtual realm, highlighting the need to study their potential carryover effects on real life.

To better understand these impacts, Ortiz de Gortari et al. (2011) introduced the concept of Game Transfer Phenomena (GTP), referring to immersive gaming’s influence on real-world behaviors, thoughts, and perceptions. Research indicates that GTP is highly prevalent, with 96.6% of players reporting experiences of GTP and 95.3% encountering it multiple times (Ortiz de Gortari & Griffiths, 2016). In some instances, GTP can pose tangible real-world risks, such as perceiving in-game visuals while driving (Ortiz de Gortari & Griffiths, 2014a; Ortiz de Gortari et al., 2015). Despite its prevalence, prior research has predominantly focused on individual differences and retrospective self-reports (Ortiz de Gortari & Griffiths, 2015), neglecting objective experiential factors, MOBA games, with their continuous action, social coordination, and intense sensory reinforcement (Mora-Cantallops & Sicilia, 2018), and provides an ideal context for studying how in-game behaviors and emotions contribute to GTP.

Assessing in-game behaviors is crucial, offering objective metrics of player achievement (Kao et al., 2008). Specifically, behaviors—like Kills, Deaths, and Assists—serve as measurable behavioral indicators of individual performance and team-based interactions (Gao et al., 2023). Meanwhile, emotional responses to gameplay—from satisfaction to frustration (Jennett et al., 2008)—may act as mediators in the persistence of gaming experiences, contributing to the transfer of cognitive and perceptual states beyond the virtual world. Since emotions shape memory and behavior (Bopp et al., 2018), understanding how post-game emotions interact with in-game behaviors to shape GTP is essential.

Accordingly, this study integrated objective in-game behavioral metrics, post-game emotional expressions derived from affective computing, and self-reported GTP experiences. A conceptual framework was developed to situate in-game behaviors, post-game emotions, and transfer dimensions within an integrated process, thereby providing conceptual guidance for both theoretical interpretation and practical application. The research enhances understanding of virtual-world immersion and experience aftereffects in MOBA gameplay while offering practical insights into game design, user experience optimization, and potential intervention strategies.

### 1.1. GTP: Definition and Research Progress

GTP is a non-volitional psychological phenomenon that describes the involuntary persistence of gaming experiences in real-world cognition, perception, and post-gameplay behavior (Ortiz de Gortari et al., 2011). The perceptual dimension of GTP involves altered perceptions, where players report seeing in-game elements superimposed on reality (Altered visual perceptions, AVP), hearing game-related sounds (Altered auditory perceptions, AAP), or feeling game-related body perception (Altered body perception, ABP) post-play (Ortiz de Gortari & Griffiths, 2014a). Cognitive aspects involve Automatic mental processes (AMP), including persistent in-game thoughts, source-monitoring errors, and involuntary decision-making adaptations shaped by gaming strategies (Ortiz de Gortari & Griffiths, 2014b). Meanwhile, Actions and Behaviors (AB) include motor actions influenced by in-game mechanics, such as attempting to interact with real-world objects as virtual elements or reacting automatically through learned responses (Ortiz de Gortari & Griffiths, 2014b).

Existing research has begun to unpack the potential impacts of virtual experiences on real-world experiences. For instance, players of highly immersive games, such as Massive Multiplayer Online games and Role-playing games, frequently report GTP due to prolonged exposure to complex interactive environments (Ortiz de Gortari et al., 2015). Although GTP is not inherently harmful, it may lead to specific safety concerns, increasing the risk of accidents. Several factors influence GTP, including age, gaming experience, session duration, and flow states. Younger players, particularly university students with higher gaming frequency and simpler routines, report higher GTP occurrence rates, whereas professional gamers may experience it less due to habituation from extensive exposure (Poels et al., 2015; Ortiz de Gortari & Griffiths, 2016). While prolonged gaming sessions have been associated with GTP occurrence rates, perceptual changes can also occur after brief gaming periods (Ortiz de Gortari & Griffiths, 2014a). Additionally, a recent study on MOBA games found a positive relationship between flow states and GTP (Gutierrez, 2021), suggesting that the highly interactive and socially immersive nature of MOBA games may facilitate GTP experiences through mechanisms.

### 1.2. In-Game Behaviors and GTP

MOBA games have achieved global popularity, marked by rising, player engagement and in-game consumption. Unlike many other game genres, MOBAs are highly social and cognitively demanding, requiring players to engage in rapid decision-making, emotional regulation, and strategic teamwork (Mora-Cantallops & Sicilia, 2018). Their intricate virtual environments foster intricate human–computer interactions, while diverse character abilities and dynamic game mechanics evoke a wide range of emotions. Additionally, the multiplayer nature of MOBA games enhances social engagement, building strong community bonds that can extend gaming experiences beyond actual gameplay.

In-game behavior in MOBA refers to the outcomes of player interactions within the game world, encompassing task completion and collaboration (Gao et al., 2023). These behaviors can be quantitatively assessed through game metrics reflecting individual performance (e.g., Kills, Deaths) and team dynamics (e.g., Assists, Assist Ratios). This study focuses on the popular MOBA game Honor of Kings (Timi Studio Group, 2015) selecting five key indicators—Gold earned (GE), Kills, Deaths, Assists, and team fight Participation Rate (PR)—were chosen as representative metrics (Rani et al., 2005). GE, Kill, Deaths reflect players’ efficiency in achieving game objectives, while Assists and PR measure cooperative gameplay and team engagement.

While some players report perceiving game-related visual overlays in real-world contexts (e.g., floating text or HUD elements) after prolonged gaming sessions (Ortiz de Gortari & Griffiths, 2014a), more recent findings suggest that such externalized intrusions are relatively uncommon. Instead, GTP tends to manifest more frequently as internal experiences, including involuntary mental imagery, altered attention, and spontaneous recall of game-related content (Ortiz de Gortari & Diseth, 2022; Szolin et al., 2023). For example, players have reported involuntary auditory imagery, such as hearing in-game music or character voice lines after gameplay (Ortiz de Gortari, 2015; Diao et al., 2024).

Previous studies have demonstrated that in-game sensory reinforcements, such as visual effects, sound cues, and interface feedback, contribute to post-game perceptual alterations. In some cases, players have also reported experiencing subtle physical sensations, such as body movements resembling in-game combat motions (Ortiz de Gortari & Griffiths, 2014a). On the other hand, players’ immersive experience in games has been identified as a key factor in the persistence of GTP (Ortiz de Gortari, 2018). Research suggests that sustained engagement and high-arousal events enhance memory retention, while interruptions and passive phases weaken immersion and perceptions of presence (Jennett et al., 2008). Additionally, King et al. (2011) found that in-game reward systems, such as earning high scores or financial incentives, enhanced player engagement and emotional intensity.

Honor of Kings uses distinct visual and auditory reinforcements for feedback. Successful eliminations trigger high-contrast visual overlays and unique kill sound effects (e.g., “Double Kill,” “Triple Kill”), while rewards are signaled with animations and sounds. These frequent and high-intensity visual and auditory stimuli heighten player attention and may persist beyond gameplay, increasing the likelihood of experiencing post-game. Conversely, Deaths disrupt immersion, forcing players into a passive respawn countdown phase with reduced sensory input. Similarly, Assists and PR reflect the level of engagement in cooperative play and presence in the virtual environment.

Beyond perception, studies suggest that the high-speed nature of certain video games, along with continual reinforcement and shifting attentional demands, can influence cognitive processing beyond gameplay (Barlett et al., 2009; Swing et al., 2010). Bailey et al. (2010) found that frequent gaming experiences reduced proactive cognitive control efficiency, hindering sustained attention on tasks and then potentially priming habitual response patterns extending beyond the game. At the motor level, involuntary actions and behaviors have been widely reported among gamers (Ortiz de Gortari et al., 2015). In the game, players who engage in repetitive screen interactions or rapid button presses may develop residual action tendencies, potentially influencing habitual gestures in real-world gestures.

On the other hand, MOBA games emphasize social interactions, making team-based dynamics fundamental to player experience. Prior research (Diao et al., 2024) found that players often perceive their ultimate goals not as individual success, but as fighting alongside teammates and fostering positive social connections. This shows that good cooperative and socially oriented in-game performance may impact cognitive and behavioral extensions beyond the virtual world.

### 1.3. In-Game Behaviors and Emotional Expression

One of the primary objectives of digital game design is to deliver rich emotional experiences through player interactions with game content, virtual characters, and other players (Sekhavat et al., 2020). Emotions critically influence the brain’s information processing and behavioral decision-making (Jin & Oh, 2022). Multiplayer games require rapid responses during social interactions with opponents and teammates, evoking diverse emotional states (Jennett et al., 2008).

Emotional expression often reflects an individual’ s feelings (Holbrook & Hirschman, 1982). Recent neuropsychological studies have revealed that distinct in-game behaviors correspond with differentiated neural activations and emotional outcomes. For instance, Shin et al. (2012) utilized EEG monitoring during CS: GO sessions and found that successful behaviors, such as killing opponents, activated regions associated with reward and positive affect, while receiving damage activated networks linked to threat detection and negative arousal. These results were further supported by self-reported data, showing that pleasant emotional states (e.g., pride, excitement) were more prevalent following high-performance segments, whereas frustration and tension increased after repeated failure or physical inaction. Similarly, Mateo-Orcajada et al. (2022) demonstrated that losses significantly increased anger, depression, and confusion among professional League of Legends players, whereas wins boosted vigor. These findings collectively suggest that emotional expressions in gaming contexts are not merely incidental reactions, but may be systematically associated with specific in-game behaviors, such as successful actions, repeated failures, or fluctuating motor patterns.

### 1.4. Emotional Expression and GTP

While the impact of emotions on behavior and cognition is widely acknowledged (Bopp et al., 2018; Peever et al., 2012), research on how players’ post-game emotional responses influence actual lives remains limited. The virtual world provides an advanced platform for human–computer interaction, where game developers utilize highly multimodal and dynamic techniques to elicit players’ emotions. Emotion theories suggest positive emotions broaden attention span, encouraging exploratory behavior, engagement, and decision-making (Fredrickson, 2001), while negative emotions narrow focus toward specific objects or environments (Kahn & Isen, 1993). In gaming, players predominantly seek positive emotional experiences (Sherry et al., 2012), often motivated by virtual enjoyment and stress relief (Diao et al., 2024). Such emotional experiences not only motivate players but also shape their ongoing interactions with the game environment.

Emotions in gaming extend beyond binary of positive and negative and are better understood as multidimensional constructs that varying in intensity and type, influencing diverse aspects of player behavior and experience (Cowen & Keltner, 2020). Recent studies, such as Brooks et al. (2024), have leveraged large-scale data and machine learning techniques to examine cross-cultural variations in vocal and facial expressions, identifying 48 distinct emotional and mental states. Emotions may vary in intensity, duration, and interaction with cognitive and social processes, shaping how players internalize and respond to game-related events. For instance, MOBA players may feel excitement after a win, frustration after a loss, pride from achieving goals, or anxiety during unexpected challenges. Additionally, team-based interactions introduce socially driven emotions, like guilt from underperforming, relief after overcoming difficult situations, or embarrassment from critical mistakes. This multidimensional perspective offers deeper insights into how emotions influence player experiences and extend beyond the virtual environment.

High-arousal emotional states during gameplay enhance memory retention and increase the likelihood of post-game cognitive and perceptual persistence (Ortiz de Gortari, 2015). For example, frustration from a loss or an intense battle sequence may lead to intrusive thoughts or involuntary mental replaying of game events. Moreover, research suggests that cathartic effects of gaming in reducing stress and anxiety (LaRose et al., 2003; Caplan, 2002), emotional responses encompass complex states beyond simple dichotomies, warranting further exploration.

## 2. Research Hypotheses

Drawing upon previous research and theoretical foundations, this study proposes that both in-game behavioral performance and emotional responses jointly contribute to the occurrence of Game Transfer Phenomena (GTP) across perceptual, cognitive, and behavioral dimensions. The specific hypotheses are as follows:

**H1.**  *In-game behavioral performance significantly predicts GTP dimensions. Specifically, successful individual performances (e.g., Kills, Gold earned) are expected to positively predict GTP dimensions, while performance failures (e.g., Deaths) are anticipated to exhibit a negative association. Cooperative behaviors (e.g., Assists, Participation rate) are hypothesized to enhance perceptual, cognitive, and behavioral transfer.*

**H2.**  *Certain post-game emotional expressions significantly predict GTP dimensions, as they reflect players’ affective responses to the match, which may extend into perceptual, cognitive, and behavioral transfers.*

**H3.**  *Post-game emotional expressions mediate the relationship between in-game behavioral performance and GTP dimensions. Behavioral performance is expected to evoke distinct emotional states that subsequently shape perceptual, cognitive, and behavioral transfers.*

## 3. Methods

### 3.1. Participants and Procedures

In this study, the popular MOBA game Honor of Kings was selected due to its widespread popularity among gamers. Participants were recruited through convenience and snowball sampling methods, with recruitment efforts promoted via the university’s online forum. All respondents were selected based on established criteria to ensure they represented hardcore players of Honor of Kings. Specifically, participants had been playing this game for at least three years, achieved a comprehensive in-game rating exceeding 75 points (at the honor king level), and played the game for a minimum of six hours per week over the past month. These criteria emphasize prolonged engagement, deep familiarity with game mechanics, and sustained time investment (Yee, 2006). The sample size was determined using G * Power 3.1, with an alpha level of 0.05, and a statistical power of 0.80. The analysis indicated that a minimum of 23 participants was required (Cohen’s d = 0.5), and the final sample of 30 participants met this requirement. Of these, 22 were male, aged between 19 and 35 years, with 22 participants aged 19–25 and 8 participants aged 26–35, 14 participants played for an average of 6–10 h per week, while 16 participants played for more than 10 h weekly.

This research was approved by the Ethics Committee, ensuring compliance with ethical standards for research involving human subjects. Participants were asked to bring their own mobile devices, following their usual gaming habits. Researchers provided a laptop equipped with an external webcam (Logitech C920e 1080p, Logitech International S.A., Lausanne, Switzerland) for facial movement capture. The experiment was conducted in a quiet, distraction-free classroom to ensure a controlled environment.

The experimental procedure included the following steps:

1. Upon arrival, participants were briefed on the study objectives, procedures, and data usage policies. They signed an informed consent form confirming their voluntary participation and right to withdraw at any time.
2. Participants engaged in a ranked match of *Honor of Kings* on their own devices. To ensure stable facial data recording without disrupting their natural gaming experience, they were instructed to maintain a seated position with unobstructed facial visibility (e.g., no hands covering the face) throughout the session. Video recording was initiated 2 min prior to the match start and continued uninterrupted for the entire duration of gameplay—this continuous recording approach was intentionally adopted to avoid any interruption to the gaming process (e.g., pausing or restarting the camera mid-session) that might distract participants.
3. At the conclusion of the match, participants were instructed to wave at the camera—a standardized gesture to mark the objective endpoint of gameplay. This gesture served two key purposes: (1) to signal the exact end time of the match for precise data segmentation, and (2) to ensure consistency in identifying the transition from gameplay to post-play states across all participants. Following the gesture, participants remained seated in the same position for a short rest period (no specific tasks assigned), during which video recording continued. The 1-min video segment immediately after the gesture was extracted for analyzing post-game facial expressions, as this period captures consolidated emotional outcomes that reflect the overall gameplay experience. In contrast, in-game emotions were not analyzed, as they are heavily influenced by fleeting, event-specific in-game occurrences and thus do not represent the holistic outcome of the session.
4. After the post-play recording, participants left the laboratory and completed an online questionnaire at their residence within 1 h of departure. The questionnaire assessed post-game experiences, including GTP and emotional responses. To preserve validity, participants were instructed not to engage in further gaming activities before completing the questionnaire, preventing new gameplay from confounding their responses.
5. Each participant received monetary compensation (10–20 RMB) for their participation.

### 3.2. Instruments

#### 3.2.1. GTP Scale

Ortiz de Gortari et al. (2015) developed the Game Transfer Phenomena (GTP) Scale, which assesses players’ experiences across five dimensions using 20 items. These dimensions cover perceptual alterations, including visual (AVP), auditory (AAP), and bodily sensations (ABP); cognitive aspects, captured by Automatic Mental Processes (AMP), such as involuntary thoughts or decision-making biases; and Actions and Behaviors (AB), referring to impulses or motor actions influenced by game mechanics. The original scale adopts a frequency-based response format (from “All the time” to “Never”) and has been widely used to measure the persistence of virtual experiences in real life.

In this study, the Chinese version of the GTP Scale was adopted (Wang et al., 2023), and adapted to fit the context of retrospective self-reporting after a single gameplay session. Based on the original structure, all 20 items and five dimensions were retained. However, two key modifications were implemented. First, to better reflect the intensity of participants’ post-game experiences, the response format was adjusted from frequency-based to agreement-based (“strongly disagree” to “strongly agree”). This semantic shift was necessary because the original frequency framework is less relevant for capturing acute reactions to a single gameplay session, whereas an agreement-based format more effectively measures the strength of immediate post-game feelings. Second, linguistic revisions were made to align item wording with the single-session context, while strictly preserving the core content of the original items. The adaptation process referenced the Wang et al. (2023) Chinese version for phrasing consistency with prior empirical constructs, with modifications limited to reframing long-term frequency to immediate post-session states (e.g., “after playing” the specific session). These adjustments do not alter the underlying constructs assessed by the GTP Scale. Instead, they improve the appropriateness of measurement for a single-session context. As a result, findings in this study reflect the intensity of participants’ immediate GTP rather than the GTP experience in longer periods. Example items from the adapted scale, with direct alignment to the original, are as follows:

“I can visualize video game images in my mind or see them with closed eyes after playing (e.g., seeing images from the game in the back of the eyelids).”

(Adapted from item 1 of the original scale: visual imagery with eyes closed)

“I can experience bodily sensations of movement as if I was in a video game after playing (e.g., lying in bed but feeling like your body or some part of your body is moving).”

(Adapted from item 5 of the original scale: bodily sensations of movement)

“I have heard the music from a game when I was not playing.”

(Adapted from item 9 of the original scale: auditory involuntary imagery)

The complete five-dimensional structure was retained, and the revised scale demonstrated excellent internal consistency in this study (Cronbach’ s α = 0.95), supporting its psychometric adequacy for measuring GTP in single-session contexts. While the agreement-based response structure may limit strict comparability with frequency-based studies, the adapted version is theoretically and empirically aligned with the original framework. Participants’ GTP scores ranged from 20 to 73, with a mean of 45.92 and a standard deviation of 15.72, suggesting that most participants experienced moderate to high levels of GTP.

#### 3.2.2. Behavioral Data and Post-Game Emotional Data

The behavioral data were automatically generated through the Honor of Kings game interface, which records a wide range of in-game metrics relevant to player performance.

The Hume AI Facial Expression Measurement model, an advanced tool in affective computing, leverages the Facial Action Coding System (FACS) 2.0 to enhance its capability in quantifying and analyzing human emotions (Cowen & Keltner, 2020; Brooks et al., 2024). Trained on millions of annotated video clips collected from diverse populations, the model benefits from extensive cross-cultural labeling, allowing robust recognition of subtle and complex facial expressions. It identifies 48 discrete emotional dimensions offering substantially greater granularity than traditional classification systems. The validity and reliability of the model have been demonstrated in recent computational affective science studies (e.g., Brooks et al., 2024), and its performance has been benchmarked against both psychological annotations and real-world expressive behavior. This tool is freely accessible to researchers via the Hume AI platform at https://www.hume.ai (accessed on 15 July 2024).

During data collection, participants’ facial videos were recorded using a standardized webcam setup under consistent lighting conditions. To ensure temporal precision, the video segments were trimmed based on pre- and post-game waving gestures to isolate the post-game affective window. From each trimmed segment, 15 frames were extracted and analyzed at a 5-frame interval. The Hume AI system computed a probability distribution across the 48 emotion dimensions for each frame and also output auxiliary metrics, such as facial length and width. These outputs were averaged per participant to create individual emotional profiles and subsequently integrated with other behavioral and survey-based indicators in the analytical model.

### 3.3. Statistical Analyses

Statistical analyses were performed using R 4.4.1 with a significance cutoff of *p* = 0.05. The relationships between GTP dimensions, behavioral factors, and post-game emotional components were primarily assessed using Mantel tests and Partial Least Squares Path Modeling (PLS-PM).

Mantel tests were employed to examine the associations between behavioral metrics and each of the five dimensions of the GTP scale. As each GTP dimension consisted of multiple questionnaire items, participants’ responses were aggregated into multi-item vectors, from which inter-individual distance matrices were computed. Similarly, emotional and in-game behavioral variables were used to construct distance matrices based on post-game emotional profiles. Mantel tests were then conducted to evaluate the correlations between these distance matrices, providing a robust approach to assess whether individuals with similar behavioral or emotional patterns also exhibited similar GTP responses. This method is particularly suitable for multidimensional psychological data and does not rely on the assumptions required by traditional parametric tests. All analyses were conducted using the “vegan” and “psych” packages (Oksanen et al., 2013; Revelle, 2017).

Following this, PLS-PM was applied to model the structural relationships among behavioral performance, emotional components, and GTP dimensions. This method offered insights into both direct and indirect pathways, quantifying the influence of behavioral and emotional variables on GTP dimensions while capturing the mediating roles of emotional components. Additionally, this approach is particularly suitable for studies with small sample sizes.

## 4. Results

This study explored the relationships between in-game behaviors, post-game emotional expressions, and the dimensions of GTP. A series of analyses, including Mantel-test correlations, principal component analysis (PCA), and Partial Least Squares Path Modeling (PLS-PM), were conducted to reveal both direct and indirect pathways underlying these associations.

### 4.1. Relationships Between Behaviors, Emotional Expressions, and GTP

#### 4.1.1. Behaviors and GTP

Mantel-test analyses were conducted to examine the relationships between players’ in-game behaviors and the dimensions of GTP (Figure S1, see Supplementary Material). The results identified significant associations between specific behavioral metrics—namely Assists and Deaths—and multiple GTP dimensions, suggesting potential links between players’ in-game actions and their post-game experiences.

Assists were positively correlated with AVP (r = 0.135, *p* = 0.030) and AMP (r = 0.146, *p* = 0.035). These findings suggest that cooperative behaviors, such as assisting teammates, are associated with both sensory and mental extensions beyond the game.

Deaths, on the other hand, were significantly correlated with AVP (r = 0.124, *p* = 0.04), AAP (r = 0.245, *p* = 0.004), and AB (r = 0.210, *p* = 0.009). These correlations indicate that more frequent in-game failures are associated with sensory (visual and auditory) and behavioral extensions in post-game experiences.

Based on these results, only in-game behaviors that demonstrated statistically significant associations with at least one GTP dimension—namely Assists and Deaths—were selected for subsequent structural path modeling.

#### 4.1.2. Post-Game Emotional Expression, In-Game Behaviors and GTP

Mantel-test analyses were conducted to examine the relationships between post-game emotional expressions and the dimensions of GTP (Figure S2, see Supplementary Material). Several significant correlations were identified, highlighting potential associations between players’ emotional states and their post-game experiences.

Specifically, AAP showed significant positive correlations with Concentration (r = 0.178, *p* = 0.032), Embarrassment (r = 0.237, *p* = 0.005), and Shame (r = 0.168, *p* = 0.037). These results suggest that auditory-related GTP dimensions are closely associated with emotions linked to cognitive and social experiences during gameplay. Similarly, AB demonstrated significant positive correlations with Disgust (r = 0.200, *p* = 0.033) and Pain (r = 0.211, *p* = 0.037), suggesting a potential relationship between behavioral extensions and aversive emotional states. However, when these GTP-related emotions were further examined in relation to in-game behavioral indices, no significant correlations were found.

To ensure analytical focus and statistical validity, only those emotional variables that demonstrated statistically significant correlations (*p* < 0.05) with at least one GTP dimension in the Mantel-test were retained for further analysis.

#### 4.1.3. Emotional Components

Based on the Mantel test results, which revealed significant associations between a subset of emotions and GTP dimensions, five emotion variables were selected as inputs for PCA. Bartlett’s test of sphericity was statistically significant (χ^2^ = 106.355, df = 10, *p* < 0.001). Following the Kaiser (1960) criterion (eigenvalue > 1), three principal components were retained, jointly explaining 97.34% of the total variance, which exceeds the commonly recommended 85% threshold. The rotated factor loading matrix is presented in Table 1, summarizing the contributions of each emotional variable to the three components.

The first component, termed Social Discomfort (SD), captures emotions related to interpersonal unease and heightened self-awareness. This dimension is defined by high loadings on Shame (0.970) and Embarrassment (0.854), highlighting the social and self-reflective nature of these emotions. The second component, labeled Aversive Reactions (AR), represents negative emotional states associated with rejection or distress. It is characterized by strong loadings on Disgust (0.988) and Pain (0.618), indicating these emotions are central to players’ negative affective responses during or after gameplay. The third component, named Cognitive Focus (CF), reflects attentional engagement and concentration. It is dominated by a significant loading on Concentration (0.956), suggesting that focused cognitive states play a prominent role in shaping emotional experiences post-gameplay.

### 4.2. Structural Relationships Among Behaviors, Emotional Expressions, and GTP

**Figure 1:**
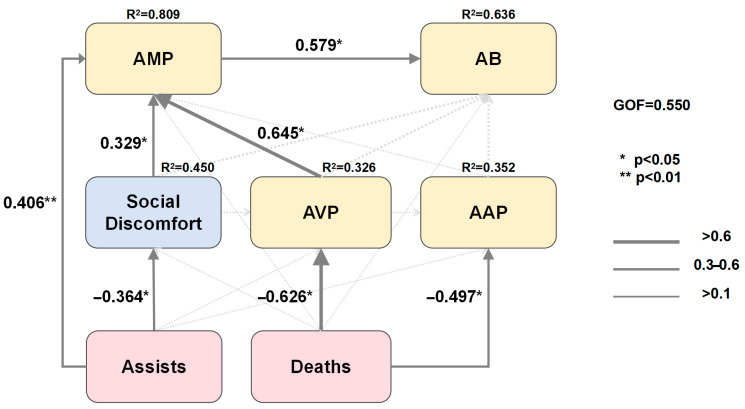
Structural pathways linking in-game behavioral, emotional influence, and GTP dimensions. Note: Arrows indicate causal relationships. Bold arrows highlight pathways with higher path coefficients, and dashed arrows represent non-significant relationships (*p* > 0.05). Non-significant variables and pathways are not shown in the figure for clarity. Statistical significance is indicated by “*” (*p* ≤ 0.05), “**” (*p* < 0.01). R^2^ values within each endogenous variable reflect the proportion of variance explained by the inner model. The overall model fit was evaluated using the GOF index (SD = Social Discomfort; AMP = Automatic Mental Processes; AB = Actions and Behaviors; AVP = Altered Visual Perceptions; AAP = Altered Auditory Perceptions).

To ensure empirical robustness, the behavior and emotion variables included in the PLS-PM model were selected based on their statistically significant correlations with GTP dimensions in the preliminary Mantel-test results. The results of the PLS-PM provided valuable insights into the relationships between in-game behavioral performance, post-game emotional expressions, and the dimensions of GTP (Figure 1). To assess the reliability and validity of the latent constructs, composite reliability (CR, i.e., Dillon–Goldstein’s ρ) and Cronbach’s alpha were calculated. All CR values exceeded the recommended threshold of 0.70, while Cronbach’s α values ranged from 0.80 to 1.00, indicating strong internal consistency. Although some constructs (e.g., Deaths, Assists, Concentration) were represented by single indicators, the remaining multi-item constructs, such as Social Discomfort (Cronbach’s α = 0.953, CR = 0.977, AVE = 0.955), AVP (Cronbach’s α = 0.826, CR = 0.885, AVE = 0.659), and AAP (Cronbach’s α = 0.863, CR = 0.932, AVE = 0.773) showed excellent reliability. The first eigenvalues of all latent constructs were substantially higher than the second, indicating unidimensionality. These results support the adequacy of the measurement model in terms of reliability and convergent validity. The overall model fit was satisfactory, as indicated by a Goodness-of-Fit (GOF) index of 0.550, reflecting robust explanatory power in capturing the interplay among these constructs.

H1 predicted that in-game behavioral performance would significantly predict GTP dimensions. This hypothesis was partially supported. Deaths had significant negative effects on AVP (β = −0.626, R^2^ = 0.326, *p* = 0.012) and AAP (β = −0.497, R^2^ = 0.352, *p* = 0.037), whereas no significant positive effects were found for Kills or Gold earned. H2 predicted that certain post-game emotional expressions would significantly predict GTP dimensions. This hypothesis was partially supported. SD emotion showed a significant direct association with AMP (β = 0.329, R^2^ = 0.809, *p* = 0.050). H3 predicted that post-game emotional expressions would mediate the relationship between in-game behavioral performance and GTP dimensions. This hypothesis was not supported. Although Assists negatively predicted SD emotion (β = −0.364, *p* = 0.047) and SD positively predicted AMP (β = 0.329, *p* = 0.050), the indirect effect along the Assists → SD → AMP pathway was not significant (ACME = 0.000, 95% CI = [0.000, 0.000], *p* = 1.000).

## 5. Discussion

The central objective of this study was to examine how in-game behavioral performance and post-game emotional expressions contribute to GTP, with the aim of uncovering the psychological mechanisms through which virtual experiences extend into real-world. In the results, four dimensions were identified as significant: Altered Visual Perceptions (AVP), Altered Auditory Perceptions (AAP), Automatic Mental Processes (AMP), and Altered Behaviors (AB). These dimensions are therefore referred to in the following discussion.

### 5.1. Empirical Implications

Regarding H1, behavioral performance was shown to influence perceptual dimensions of GTP, although not in the anticipated direction for all indicators. Within the PLS-PM model, Deaths negatively predicted AVP and AAP, indicating that frequent in-game failures attenuate perceptual transfer. Such outcomes suggest that failure-related experiences may disrupt sensory immersion through frustration or disengagement, thereby reducing the likelihood of perceptual residues after play. In addition, the design features of Honor of Kings may have contributed to this attenuation: death animations typically involve a grayscale overlay, temporary interface deactivation, and subtle sound effects, which collectively reduce the salience and memorability of perceptual cues. In contrast, Assists, reflecting collaborative team dynamics, were positively associated with AMP. This indicates that cooperative gameplay enhances cognitive retention, reinforcing the role of social collaboration in the transfer of virtual experiences into real-world cognitive processes (Tauer & Harackiewicz, 2004). Teamwork fosters strategic coordination, mutual support, and shared accomplishments, potentially strengthening automatic mental processes after gameplay (e.g., persistent game-related thoughts, attentional biases toward in-game cues, or misattributions between virtual and real-world interactions). These findings extend existing literature on the motivational and immersive benefits of teamwork (Tauer & Harackiewicz, 2004; Pe-Than et al., 2015), emphasizing that cooperative dynamics not only drive in-game success but also facilitate the transfer of virtual experiences into real-world mental domains.

Regarding H2, Social Discomfort was significantly associated with AMP, indicating that post-game affective states play a role in cognitive transfer. The broader emotional constructs analyzed—Social Discomfort, Aversive Reactions, and Cognitive Focus—enrich the understanding of how emotional complexity shapes experience transfer. Moving beyond the traditional dichotomy of positive versus negative emotions (Tang et al., 2024), the present findings emphasize the multidimensional role of emotions in post-game phenomena. Specifically, Social Discomfort, was closely linked to AMP. These socially oriented emotions frequently arise in response to perceived failures or deviations from group expectations, and may elicit automatic mental processes causing involuntary, repetitive thoughts about the game after playing. Embarrassment, as noted by Babcock and Sabini (1990) and Keltner (1996), typically results from minor lapses or public mistakes, whereas shame, more intense, often relates to personal/social failure, especially in competition (Tracy & Matsumoto, 2008). In multiplayer games, these emotions may be triggered not only by visible in-game errors but also by a perceived lack of contribution to the team’s success. Players who internalize such perceptions may experience strong automatic mental processes, enhancing cognitive processing and affective recall. Importantly, while Social Discomfort is conventionally regarded as maladaptive, this study points to its potential in facilitating mental extension. Such emotions may function as affective cues that prompt deeper cognitive elaboration and self-regulation, in line with Kahn and Isen’s (1993) argument that certain negative emotions narrow more attention for focused processing.

Regarding H3, the hypothesis that post-game emotional states would mediate the relationship between in-game performance and GTP dimensions was not supported in the PLS-PM model. Interestingly, Mantel-test results revealed significant correlations between AAP and emotions. This dissociation may be attributed to the modality-specific structure of game feedback in *Honor of Kings*. Auditory elements, such as skill-trigger sounds, kill streak announcements, and dynamic background music—remain continuously active throughout and immediately after gameplay, persisting even during post-game. In contrast, visual feedback is spatially constrained, often standardized across matches, and can be selectively ignored. Notably, unpleasant emotions may lead to visual avoidance, but audio cues persist through headphones or device speakers, maintaining emotional presence. This finding suggests that auditory design may play a more active role in reinforcing emotion-related experiential residues, warranting further cross-genre exploration.

Moreover, cooperative performance indicators provided additional insights. Assists were negatively associated with post-game emotional states characterized by Social Discomfort, such as shame and embarrassment, suggesting that collaborative gameplay may buffer players against self-conscious negative emotions typically triggered by competitive contexts. In addition, AMP was positively associated with AB, and AVP also showed a positive link to AMP, indicating that players with strong sensory engagement and cognitive involvement during gameplay may be more likely to extend such immersive effects into post-game behavioral patterns. This finding is consistent with prior research emphasizing the co-activation of sensory immersion and attentional focus in sustaining prolonged post-game impacts (Daassi & Debbabi, 2021; Zhang & Song, 2022). Taken together, these results suggest that while the hypothesized mediation pathways were not supported, alternative parallel or reinforcing processes may underlie the interplay between performance, emotions, and transfer outcomes.

### 5.2. Conceptual Framework

**Figure 2:**
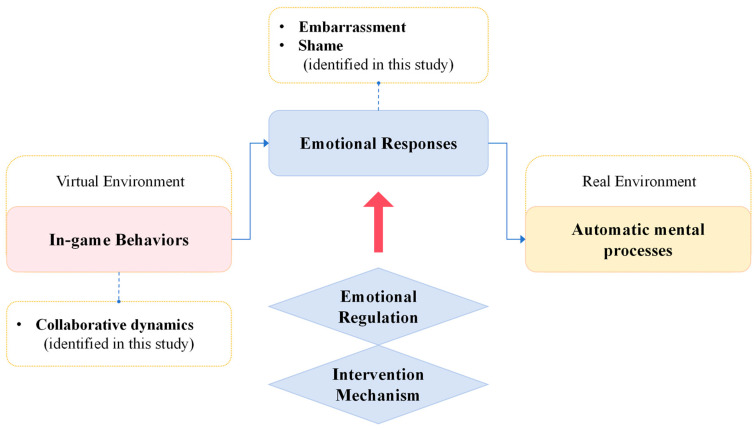
Conceptual framework for transfer in MOBA game environments. Solid arrows represent the directional flow or process. Dashed lines indicate an associative or belonging relationship. The red arrow points to the proposed intervention.

Based on findings, the conceptual framework shown in Figure 2 highlights a critical pathway identified in this study: negative collaborative dynamics in MOBA virtual environments can elicit specific emotional responses, notably embarrassment and shame, which were empirically observed in our data. These emotions, in turn, appear to trigger automatic mental processes that extend into players’ real-world cognitive patterns. Although AMP are not inherently negative, their emotional outcomes may modulate persistent in-game thoughts and involuntary decision-making adaptations shaped by gaming strategies after exiting the game environment. The objective of post-game regulation is therefore not to suppress automaticity itself, but rather to modulate the emotional valence that drives these processes, particularly when they are fueled by discomfort emotions. To address this process proactively, the integration of structured emotional regulation strategies into post-game interventions is recommended. Specifically, implementing cognitive reappraisal techniques may enable players to reconstruct the emotional meaning of in-game collaboration, thereby reducing the likelihood of discomfort emotional spillover into real-world contexts.

This approach draws upon the fourth category of the emotion regulation process—cognitive change as outlined by Gross (2015), which emphasizes modifying the meaning of emotional stimuli to alter their psychological impact. In practical terms, this could involve post-match interactive prompts (e.g., “Your teamwork helped secure key moments” or “You supported your team when it mattered most”) to help reframe the emotional interpretation of collaborative actions. By fostering a sense of purpose and contribution through team-based roles, such interventions may mitigate socially discomfort emotions and prevent their spillover into real-world interactions. Future research should aim to operationalize and empirically evaluate the effectiveness of these strategies in regulating post-game emotional responses and cognitive extensions.

## 6. Conclusions and Limitations

This study provides an examination of the relationships between in-game behaviors, post-game emotional expressions, and GTP, offering both theoretical and practical insights into how MOBA gaming experiences extend into real-world cognition, perception, and behavior. By integrating behavioral metrics, emotional dimensions, and GTP’s components, the findings highlight the nuanced interaction between individual and team dynamics, emotional states, and their carryover effects.

The results underscore the critical roles of personal performance (e.g., Deaths) and collaborative dynamics (e.g., Assists) in shaping players’ post-game experiences. Specifically, repeated individual death was associated with reducing in certain perceptual dimensions of GTP, and collaborative behaviors, such as providing assists, were positively related to the activation of automatic mental processes, suggesting that teamwork may facilitate the persistence of cognitive routines beyond the game context. Importantly, the study identified social discomfort emotions as significant affective correlates of these processes. These emotions, which frequently arose in negative collaborative dynamics, appear to intensify the likelihood of players experiencing involuntary game-related thoughts and behaviors after gameplay.

Based on these insights, the study recommends the integration of structured emotional regulation strategies (cognitive reappraisal) into post-game interventions. Practically, the study offers actionable recommendations for game developers, psychologists, and user experience designers to design interventions that balance the entertainment value of gaming environments with the need to address potential unintended transfer effects.

Despite its contributions, this study has several limitations. To assess the robustness of the model, a bootstrap procedure with 1000 resamples was conducted. While several direct effects (e.g., Deaths → AVP: β = −0.626, 95% CI [−1.023, −0.123]; Deaths → AAP: β = −0.497, 95% CI [−0.847, −0.010]) remained statistically significant, some indirect or mediational paths exhibited wide confidence intervals crossing zero, indicating potential estimation instability. Post hoc power analysis indicated that only the AMP pathway demonstrated sufficient statistical power (power ≈ 0.99), whereas all other key constructs showed inadequate power (all power < 0.21). This suggests that future studies with larger samples are therefore needed to enhance the statistical reliability of indirect pathway estimations and validate the observed model structure. Expanding the sample size may also allow future research to enrich understanding of the emotional mechanisms underlying GTP.

Moreover, although this study examined GTP using a validated self-report instrument, it relied entirely on participants’ retrospective evaluations. Such reliance introduces potential biases associated with memory recall and subjective interpretation. To strengthen construct validity and reduce these biases, integrating more objective measures should be further considered, such as eye movement, gameplay log analysis, EEG analysis.

Finally, while the study examines the occurrence of GTP, it does not specify which in-game elements (e.g., visual symbols, motor actions, narrative prompts) are most responsible for driving transfer experiences. This limits our understanding of the mechanisms by which virtual experiences shape post-game responses. Future research should extend this framework by identifying and isolating specific game elements that are more likely to be transferred, in order to map their influence on players’ real-world perceptions and behaviors more precisely.

## Figures and Tables

**Table 1:** Rotated Component Matrix.

Emotion	SD	AR	CF
Shame	0.970		
Embarrassment	0.854		
Pain		0.618	
Disgust		0.988	
Concentration			0.956

## Data Availability

The data presented in this study are available on request from the corresponding author. The data are not publicly available due to privacy and ethical restrictions.

## Supplementary Materials

The following supporting information can be downloaded at: https://www.mdpi.com/article/10.3390/bs15091203/s1, Figure S1. Analysis of the correlations between in-game behaviors and GTP. Figure S2. Analysis of the correlations between post-game emotional expressions and GTP.
