# Supplementary material for: The Transfer of In-Game Behaviors and Emotions to Real-World Experiences in Game World
Source: Behav Sci (Basel). 2025 Sep 4;15(9):1203. doi: 10.3390/bs15091203 (PMC12466803; doi:10.3390/bs15091203)
Supplement: Supplementary file 1 [file behavsci-15-01203-s001.zip › behavsci-3720031-supplementary.pdf]

Supplementary Materials

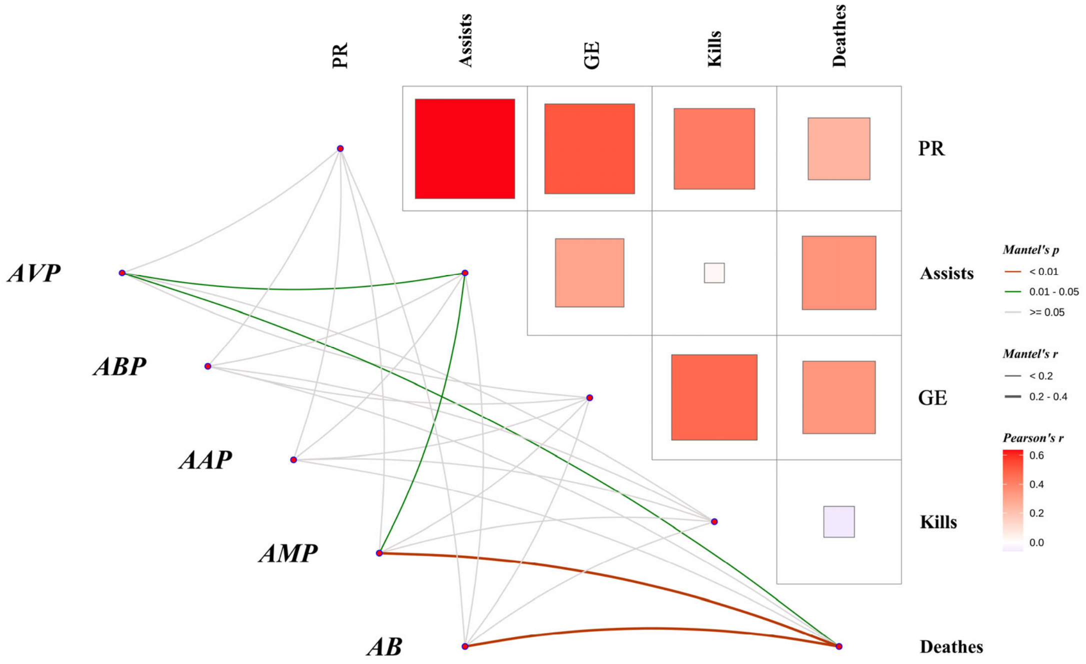

Figure S1. Analysis of the correlations between in-game behaviors and GTP

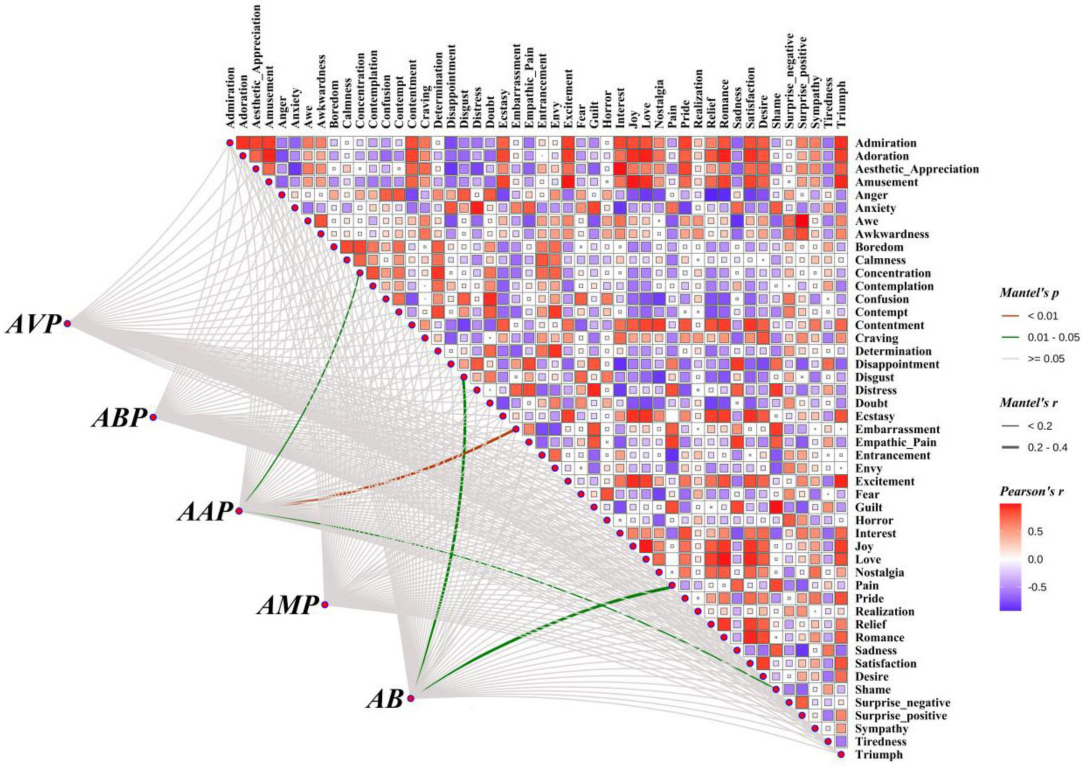

Figure S2. Analysis of the correlations between post-game emotional expressions and GTP
